# Enhancing Mechanical and Impact Properties of Flax/Glass and Jute/Glass Hybrid Composites Through KOH Alkaline Treatment

**DOI:** 10.3390/polym17060804

**Published:** 2025-03-18

**Authors:** Sultan Ullah, Arvydas Palevicius, Giedrius Janusas, Zeeshan Ul-Hasan

**Affiliations:** 1 Department of Mechanical Engineering, Faculty of Mechanical Engineering and Design, Kaunas University of Technology, Studentų 56, LT-51424 Kaunas, Lithuania; arvydas.palevicius@ktu.lt (A.P.); giedrius.janusas@ktu.lt (G.J.); 2 Department of Materials Engineering, School of Engineering & Technology, National Textile University, Sheikhupura Road, Faisalabad 37610, Pakistan; zeeshanaltaf77@gmail.com

**Keywords:** hybrid composites, potassium hydroxide (KOH) treatment, mechanical properties, fiber–matrix adhesion, SEM analysis, sustainable materials

## Abstract

This research investigates the influence of potassium hydroxide (KOH) treatment on the mechanical, flexural, and impact properties of flax/glass and jute/glass hybrid composites. Hybrid composite materials have been developed, incorporating natural fibers that are both treated and untreated by KOH, with glass fiber within an epoxy matrix. Natural fibers, such as flax and jute, were chemically treated using different KOH concentrations and immersion times specific to each fiber type. Following the treatment, both fibers were rinsed with distilled water and subsequently dried. The natural fiber’s chemical interaction was analysed using FTIR. Hybrid composites were fabricated via the integration of intercalated layers of natural fibers and glass fiber using hand layup followed by compression molding. Mechanical properties, including impact resistance, flexural strength, elastic modulus, and tensile strength, were evaluated in accordance with ASTM guidelines. KOH-treated flax/glass composites (T-F2G2) demonstrated enhanced fiber–matrix bonding, indicated by elevated tensile strength (118.16 MPa) and flexural strength (168.94 MPa) relative to untreated samples. The impact strength of T-F2G2 composites increased to 39.33 KJ/m^2^ due to the removal of impurities and exposure of hydroxyl groups, which interact with K^+^ ions in KOH, thereby improving their mechanical properties. SEM analysis of cracked surfaces confirmed enhanced bonding and reduced fiber pull-out, indicating improved interfacial compatibility. The findings demonstrate that KOH treatment effectively preserves cellulose integrity and enhances fiber–matrix interactions, positioning it as a viable alternative to NaOH for hybrid composites suitable for lightweight and environmentally sustainable industrial applications.

## 1. Introduction

Natural fiber composites are attracting significant interest as viable alternatives to synthetic composites owing to the growing need for eco-friendly materials. Natural fibers such as flax, jute, and hemp are ecologically sustainable and recyclable, as they originate from renewable resources. In alignment with global efforts to mitigate environmental impact, these fibers possess a lower carbon footprint compared to their synthetic counterparts. Furthermore, they are appropriate for use in the automotive, construction, and packaging sectors, where lightweight and high-performance materials are critical due to their low density, elevated specific strength, and rigidity [1,2,3]. Their abundance and regenerative capacity render them suitable for extensive industrial application, enhancing their cost-effectiveness [4]. However, certain challenges hinder the widespread utilisation of natural fiber composites. Surface impurities, such as those involving lignin, hemicellulose, pectin, and waxes, are inherently found in the fibers and inhibit their strong adhesion to polymer matrices [5]. Furthermore, due to their hydrophilic nature, they absorb moisture, resulting in swelling, debonding, and dimensional instability of the composite material [6]. Hydrophilic fibers and hydrophobic polymer matrices exhibit incompatibility, leading to weak adhesion and compromised mechanical stability of the composite [7]. The relatively low thermal stability of natural fibers compared to synthetic alternatives limits their application in high-temperature environments, and variations in cultivation, harvesting, and processing further affect fiber quality [8,9]. Research has been conducted on surface modification techniques to improve the compatibility of natural fibers with polymer matrices to address these limitations. Alkali treatments are commonly employed to enhance fiber–matrix adhesion by augmenting surface roughness and removing impurities, particularly with the application of sodium hydroxide (NaOH) [10]. Introducing NaOH at elevated concentrations can compromise the structural integrity of cellulose in fibers, hence diminishing their mechanical characteristics and tensile strength [11]. Potassium hydroxide (KOH), a milder alkali treatment, has attracted attention as a more effective alternative. Research demonstrates that KOH-processing successfully removes impurities while maintaining cellulose integrity and improves fiber–matrix adhesion with minimal degradation, resulting in better the mechanical attributes [12,13,14]. The production of hybrid composites that integrate natural fibers (flax and jute) with manmade reinforcements, such as glass fibers, has demonstrated efficacy in overcoming these problems. Hybrid composites can attain an appropriate ratio of the advantages of organic fibers, including being lightweight and contributing to conservation efforts, and in terms of the enhanced mechanical and heat capabilities of synthetic counterparts by the incorporation of manmade materials [15,16]. Hybrid composites have attracted considerable interest because they effectively merge the benefits of different fiber types, enhancing mechanical properties, durability, and impact resistance [17]. The hybrid mechanism facilitates rapid stress transfer among fibers, thereby minimising premature failure and improving energy absorption during impact [18]. Research conducted by Xian et al. [19] demonstrates that hybrid composites possess enhanced fatigue performance, rendering them appropriate for structural applications. Furthermore, hybrid effects, including positive hybridisation, raise damage tolerance and resistance to degradation of the environment [20]. Barjasteh et al. [21] demonstrated that hybridisation enhances durability properties, especially under moisture and thermal aging conditions, by optimising the strengths and weaknesses of individual fibers. Integrating processed organic fibers with glass constituents considerably enhances breaking strength, bending strength, and resistance to the impact of material composites, making it suitable for various usages in engineering [22]. Biochemical processing of cellulose fibers is crucial in influencing the functionality of hybrid composites, especially in functions demanding lightweight and durable substances [23]. Many investigations have examined the possibilities of natural fibers and their hybrid composites. Researchers have highlighted the ecological advantages of natural fibers, while acknowledging difficulties such as moisture retention and variable characteristics [24]. An alkaline-method-enhanced fiber–matrix interaction also revealed potential consequences of the use of NaOH, including cellulose breakdown at elevated quantities [25]. KOH is an induced treatment (not intensive), which serves as a substitute for treatment with NaOH, with KOH-treated fibers demonstrating improved stress and bending capabilities while preserving their dimensional stability [26]. Alkali treatment using NaOH and KOH markedly improves the mechanical and impact properties of composites formed from natural fibers. This enhancement occurs through the removal of non-cellulosic impurities, an increase in texture of the surface, and an improvement in fiber–matrix adherence [27]. This leads to enhanced tensile strength, flexural strength, and impact resistance, as fibers demonstrate improved transfer of force performance and less fiber removal [28]. Although NaOH treatment has been extensively examined, recent investigations underscore that KOH treatment presents a gentler alternative, successfully improving interfacial bonding while reducing fiber degradation [29]. Research indicates that composites treated with alkali exhibit enhanced energy absorption and better resilience [30]. Enhancements in stiffness are associated with fiber swelling, the exposure of hydroxyl (–OH) groups, and improved stress distribution, resulting in increased toughness and resistance to crack propagation [11]. Moreover, hybrid composites that integrate both organic and manmade fibers have enhanced mechanical efficiency, especially for tensile strength and durability against impacts [31]. Scientists examined the microstructural functioning of processed composite fibers and recognised the significance of surface treatments in alleviating failure modes, including delamination and fiber pullout [32].

This research seeks to create lightweight hybrid materials by integrating KOH-treated natural fibers (flax and jute) with glass fibers within an epoxy resin network. The objective is to evaluate the mechanical performance of these hybrid composites, focusing on tensile strength, flexural strength, and impact resistance, to assess the effectiveness of KOH treatment in enhancing fiber–matrix bonding. Despite extensive studies on NaOH treatment for improving fiber–matrix adhesion in natural fiber composites, research on KOH treatment for hybrid composites, particularly flax/glass and jute/glass systems, remains limited. While NaOH is known for its effectiveness, it often leads to excessive fiber degradation. The impact of KOH treatment on the mechanical, flexural, and impact properties of hybrid composites has not been widely explored, leaving a gap in understanding its potential as a viable alternative for enhancing composite performance while preserving fiber integrity. Our research also includes an investigation of the composites’ microstructural behaviors using scanning electron microscopy (SEM) to analyse failure mechanisms such as fiber pullout and delamination. By addressing the challenges associated with natural fibers and exploring their integration into hybrid composites, this study seeks to contribute to the advancement of sustainable, high-performance materials for engineering applications. The future direction of this research involves exploring other surface modification techniques and hybridisation approaches to further enhance the mechanical and thermal performance of natural fiber composites for use in more demanding applications.

## 2. Materials and Methods

### 2.1. Materials

In this study, plain jute and flax fabrics were utilised as natural reinforcement materials, while synthetic glass fabric was incorporated to fabricate hybrid composites. The natural fibers underwent alkaline treatment using potassium hydroxide (KOH) flakes with a concentration of 98 ± 1%, supplied by Pakistan Chemical. Some of the physical and mechanical properties of natural fibers (flax and jute), and synthetic fiber (glass) are summarised in Table 1, Table 2, and Table 3, respectively.

The matrix material used for composite fabrication was a thermosetting epoxy resin identified as Araldite LY 556 with a density of 1.2 g/cm^3^, procured from Samaro, Beynost, France, and the properties are shown in Table 4. To cure the epoxy resin, standard hardener was utilised as the catalyst supplied by Sigma-Aldrich, St. Louis, MO, USA. Epoxy resin was chosen due to its superior mechanical properties, strong fiber adhesion, and durability. While both epoxy and natural fibers absorb moisture due to hydroxyl groups, epoxy ensures better interfacial bonding, enhancing load transfer and composite strength. In contrast, hydrophobic thermoplastics offer moisture resistance but weaker fiber adhesion, making them less suitable for structural applications.

### 2.2. Alkaline Treatment of Natural Fabrics/Reinforcements

**Figure 1:**
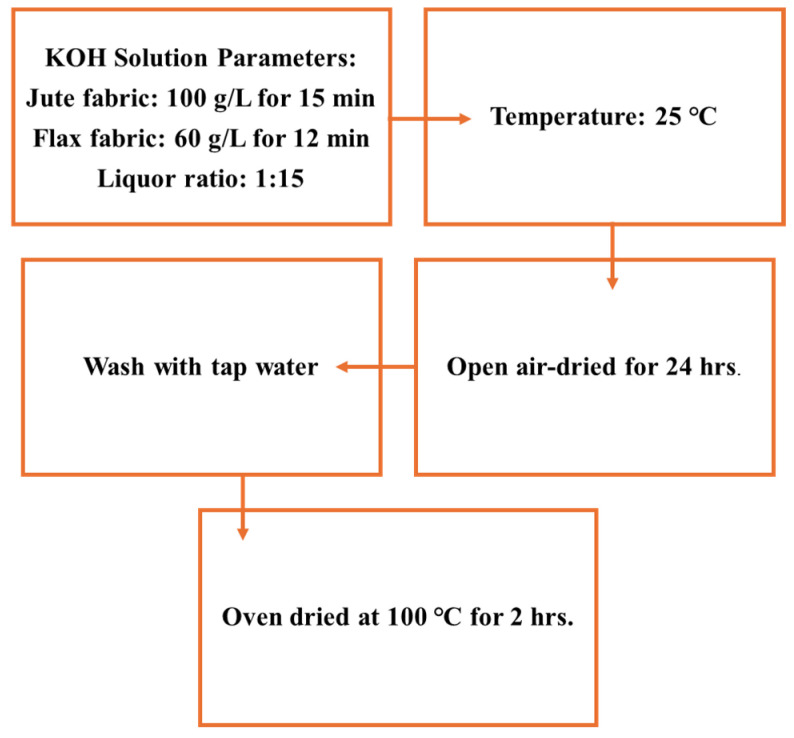
Scheme for KOH alkalisation.

Jute fabric was treated with a 100 g/L KOH solution at 25 °C with a fabric-to-liquor ratio of 1:15 for 15 min, while flax fabric underwent a similar treatment with a 60 g/L KOH solution for 12 min, maintaining the same ratio and temperature. The selected fabric-to-liquor ratio, KOH concentration, and treatment duration were optimized to enhance fiber–matrix adhesion without compromising the fibers’ structural integrity [11,33,34,35,36,37,38,39,40,41]. The treated fabrics were thoroughly washed under running tap water to remove any residual KOH from the natural fibers (jute and flax). The washing process continued until the fibers reached a neutral pH of 7, which was confirmed using litmus paper. After washing, the natural fabrics (reinforcement materials) were air-dried under open atmospheric conditions for 24 h, followed by oven drying at 100 °C for 2 h to ensure complete removal of moisture. Once dried, the fabrics were stored in separate sealed bags to maintain their condition for subsequent use. The overall alkalisation process is illustrated in Figure 1.

KOH treatment effectively modifies the fiber surface by removing non-cellulosic impurities such as lignin, hemicellulose, pectin, and waxes, leading to enhanced fiber–matrix adhesion. This process disrupts ester bonds in hemicellulose and saponifies surface waxes, exposing hydroxyl (–OH) groups that improve wettability and hydrogen bonding with the epoxy matrix. Unlike NaOH, KOH is a milder alkaline agent, minimising cellulose degradation while enhancing surface roughness, thereby preserving fiber integrity and improving mechanical interlocking with the matrix.

### 2.3. Fabrication of Hybrid Composites

**Figure 2:**
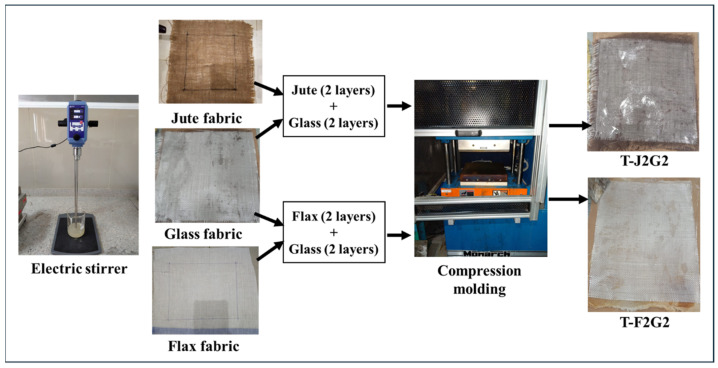
Scheme of hybrid composites fabrication.

Four-ply laminated composite slabs of both untreated and treated glass/jute and glass/flax were fabricated using the hand lay-up technique followed by compression molding. The reinforcement materials, including glass, jute, and flax fabrics, were cut into dimensions of 304 × 304 mm. and arranged in a stacking sequence of [0/90/90/0] on a metallic mold plate. To facilitate easy removal of the composite after curing, a polyamide film was used as a release layer. The epoxy resin and hardener were mixed thoroughly in a recommended ratio of 5:1 to ensure a uniform distribution of the hardener in the resin. The resulting mixture was evenly applied with a roller between the fabric layers to achieve complete wetting of the reinforcements. The laminated stack was then subjected to compression molding, applying a pressure of 14.22 bar at a temperature of 120 °C for 40 min to cure the composite as shown in Table 5. This fabrication method ensured good fiber–matrix bonding and uniform laminate quality, making the composites suitable for subsequent mechanical and structural testing. The fabrication scheme is shown in Figure 2.

### 2.4. Characterisation of Fabrics/Reinforcements

#### 2.4.1. Fourier Transform Infrared Spectroscopy (FTIR)

FTIR spectra for both untreated and KOH-treated flax and jute fabrics were obtained using IR Tracer 100 spectroscope (Shimadzu, Columbia, MD, USA), with a wavenumber ranging from 500 to 4000 cm^−1^.

#### 2.4.2. Mechanical Testing and Characterisation

Both jute and flax fabrics, before and after KOH-alkaline treatment, were evaluated for various physical and mechanical properties. Yarn count was determined following ASTM D1059-17, while ends and picks per inch were assessed using ASTM D3775-17. Fabric tensile strength was measured using the strip test method in accordance with ASTM D5035-11 (2019), and the fabric weight in grams per square meter (GSM) was determined following ASTM D3776. The tensile tests were conducted using the Textile Tensile Strength Tester (Model KG-300) manufactured by DAIEI KAGAKUSEIKI, Kyoto, Japan. Furthermore, fabric shrinkage, both pre- and post-washing, was assessed according to ISO 5077 utilising the Launder Meter L-8, produced by DAIEI KAGAKUSEIKI, Japan. These standardised methodologies guaranteed uniform and precise description of the materials for subsequent examinations.

### 2.5. Evaluation and Testing of Composites

#### 2.5.1. Tensile Testing

Tensile testing for both modified (T-J2G2, T-F2G2) and unprocessed (J2G2, F2G2) specimens was conducted in accordance with the ASTM D3039 standard utilising the Zwick Roell Z100 Universal Testing Machine (UTM), Ulm, Germany. The samples were fabricated with dimensions of 200 × 25 × 1 mm for the flax/glass samples and 200 × 25 × 2 mm for the jute/glass samples, respectively, with a gauge length of 50 mm established between the grips. The tests were conducted at a crosshead speed of 2 mm/min, and the average results from five specimens were documented and presented.

#### 2.5.2. Flexural Testing

The bending test for all composite specimens was performed in accordance with the ASTM D7264 standard. The test specimens were prepared with dimensions of 120 × 13 × 1 mm for flax/glass composites, and 120 × 13 × 2 mm for jute/glass composites. A gauge length of 50 mm was established, and measurement was performed at a crosshead velocity of 1 mm/min. The bending characteristics, encompassing stress–strain behavior, flexural strength, and the modulus of the composites, were examined and deliberated upon. The results are derived from the mean values of five specimens for each composite, guaranteeing reliability and precision in the provided data.

#### 2.5.3. Impact Testing

The resistance to impact of the materials was assessed per ISO 179-1, utilising a pendulum impact tester (model HIT50P) with an impact energy of 5 J. The test specimens were prepared with dimensions of 100 × 10 × 1 mm for flax/glass composites and 100 × 10 × 2 mm for jute/glass composites. The experiments were performed without a notch to evaluate the unnotched impact characteristics of the materials. Five specimens were evaluated for each composite, and the mean values were documented and presented to guarantee consistency and reliability in the findings.

#### 2.5.4. Scanning Electron Microscopy (SEM)

The microscopic characteristics of the broken samples from tensile, bending, and impact tests for the untreated and treated samples were examined using a scanning electron microscope (Hitachi S-3400N, LEI, Kauno, Lithuania). This investigation was performed to investigate the failure processes and acquire insights into the behavior of the composites under various loading circumstances.

## 3. Results and Discussions

### 3.1. Characterisation of Composite Preform

#### 3.1.1. Physical and Tensile Properties of Flax and Jute Fabrics

Table 2 provides yarn density, GSM, and shrinkage percentages for both flax and jute fabrics. F-BT and F-AT represent flax fabric before and after treatment, respectively. Similarly, J-BT denotes jute fabric before treatment, while J-AT refers to jute fabric after treatment, in Table 6.

**Figure 3:**
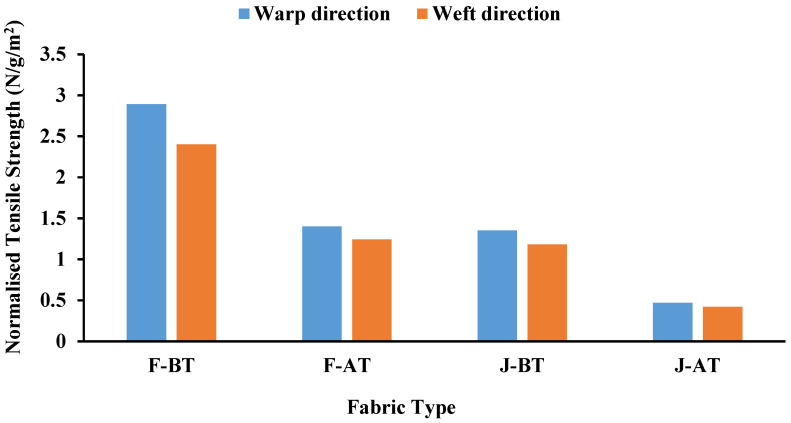
Normalised tensile strength of untreated (F-BT, J-BT) and treated (F-AT, J-AT) flax and jute fabrics.

After alkaline treatment, both jute and flax fabrics exhibited significant shrinkage, leading to higher thread density and GSM. For example, the warp and weft shrinkage percentages for flax fabric (F-AT) decreased to 0.55% and 0.45%, respectively, from 4.43% and 3.04% for untreated flax fabric (F-BT). Similarly, jute fabric (J-AT) showed reduced shrinkage percentages of 0.35% (warp) and 0.27% (weft) compared to 5.00% (warp) and 2.14% (weft) for untreated jute fabric (J-BT). This aligns with reports that alkaline treatment removes lignin, hemicellulose, and waxes, enhancing fiber-packing density and reducing inter-fiber spacing. As a result, the GSM increased from 204 g/m^2^ to 210 g/m^2^ for flax fabric and from 233 g/m^2^ to 315 g/m^2^ for jute fabric due to shrinkage-induced compaction. However, this treatment caused a decrease in tensile strength, as observed in flax fabric (F-AT) with ultimate tensile strengths (UTS) of 294.3 N (warp) and 259.97 N (weft) compared to 588.6 N (warp) and 490.5 N (weft) for untreated flax fabric (F-BT). Similarly, tensile strength for treated jute fabric (J-AT) was 147.15 N (warp) and 132.44 N (weft), lower than untreated values of 313.92 N (warp) and 274.68 N (weft). These reductions are attributed to the removal of cementing materials and changes in the crystalline structure of cellulose, where conversion from cellulose type I to type II increases amorphous regions and weakens fiber structure. Additionally, the normalized tensile strength of treated fabrics was lower than untreated ones, as shown in Figure 3, confirming that while KOH treatment improves fiber–matrix adhesion, it compromises fiber integrity due to structural changes [42,43,44]. These findings highlight the trade-offs in using alkaline treatment to enhance surface properties for composite fabrication. Furthermore, the normalised tensile strength (N) is determined using the formula provided in Equation (1):

(1) Normalised tensile strength=Tensile strengthAreal density

#### 3.1.2. FTIR Analysis

Spectrophotometric research was performed to assess the impact of KOH-induced alkaline conditions on flax and jute fibers, which consist mostly of lignin, cellulose, hemicellulose, pectin, and waxes. The procedure sought to diminish moisture absorption, improve interfacial adhesion with the matrix by eliminating non-cellulosic parts, limit distortion, and maintain the cell wall network. Potassium hydroxide efficiently eliminates contaminants like lignin, hemicellulose, pectin, and waxes, thereby revealing hydroxyl (–OH) groups on the cellulose surface. This enhances fiber–matrix bonding by improving surface roughness and enabling hydrogen bond formation. Additionally, the saponification of waxes and breaking of ester bonds cleans the fiber surface, increasing compatibility with polymer matrices and contributing to improved adhesion and mechanical performance in composites.

**Figure 4:**
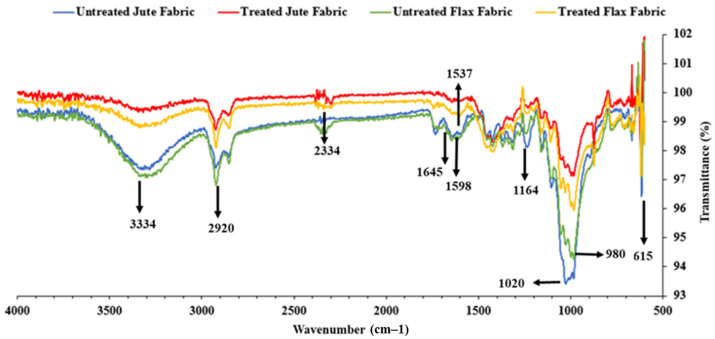
FTIR spectra of untreated and treated jute and flax fabrics.

The FTIR spectrum of untreated and KOH-treated flax and jute fabrics is shown in Figure 4, highlighting the chemical changes caused by the treatment. The broad peak at approximately 3334 cm^−1^ corresponds to –OH stretching, representing hydroxyl groups in cellulose, hemicellulose, and lignin. A reduction in this peak in treated fabrics indicates the removal of non-cellulosic components, exposing the cellulose structure. The peak at 2920 cm^−1^, associated with aliphatic C–H stretching, shows decreased intensity after treatment, suggesting the removal of surface waxes and hemicellulose.

The peak observed at 2334 cm^−1^ reflects the presence of residual impurities, with variations attributed to structural changes caused by KOH treatment. Peaks at 1645 cm^−1^ and 1598 cm^−1^, linked to C=O stretching in lignin and pectin, confirm their removal, while the disappearance of the peak at 1537 cm^−1^, associated with aromatic ring vibrations in lignin, indicates lignin breakdown. A reduction in the peak at 1164 cm^−1^, related to C–O–C stretching in cellulose and hemicellulose, reflects partial hemicellulose removal, while the preserved intensity indicates that the cellulose structure remains intact.

The sharp peak at 1020 cm^−1^ represents C-O stretching in alcoholic bonds, indicating greater exposure of cellulose after impurities were removed. Peaks at 980 cm^−1^ and 615 cm^−1^ correspond to out-of-plane vibrations and deformation modes of cellulose, further confirming structural modifications. Overall, the FTIR spectrum demonstrates that KOH treatment effectively cleans the fiber surfaces by removing impurities, such as lignin, hemicellulose, and waxes, while preserving the cellulose backbone [45,46,47]. These changes enhance the surface properties of flax and jute fabrics, improving their compatibility with polymer matrices in composite applications.

### 3.2. Characterisation of Composites

#### 3.2.1. Tensile Properties

**Figure 5:**
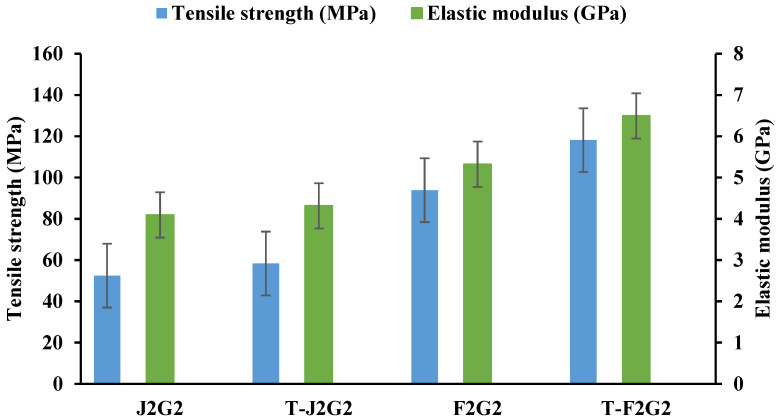
Graph showing ultimate tensile strength and elastic modulus of treated and untreated hybrid composites.

The ultimate tensile strength (MPa) and elastic modulus (GPa) of hybrid composites comprising flax/glass, and jute/glass reinforcements, both untreated and KOH-treated, are illustrated in Figure 5. Untreated jute/glass composites (J2G2) exhibit a lower tensile strength compared to untreated flax/glass composites (F2G2), with values around 52.5 MPa and 93.87 MPa, respectively. KOH-treated jute/glass composites (T-J2G2) show a slight improvement in tensile strength compared to their untreated counterpart, increasing to approximately 58.35 MPa. For KOH-treated flax/glass composites (T-F2G2), tensile strength increases significantly to about 118.16 MPa, highlighting the superior compatibility and bonding achieved through KOH treatment. The elastic modulus of the composites follows a similar trend to tensile strength. Untreated flax/glass (F2G2) and jute/glass (J2G2) composites exhibit lower elastic modulus values of around 5.33 GPa and 4.09 GPa, respectively. KOH treatment enhances the modulus for both flax/glass and jute/glass composites, with T-F2G2 achieving the highest value of 6.5 GPa, reflecting improved stiffness due to better fiber–matrix adhesion.

**Figure 6:**
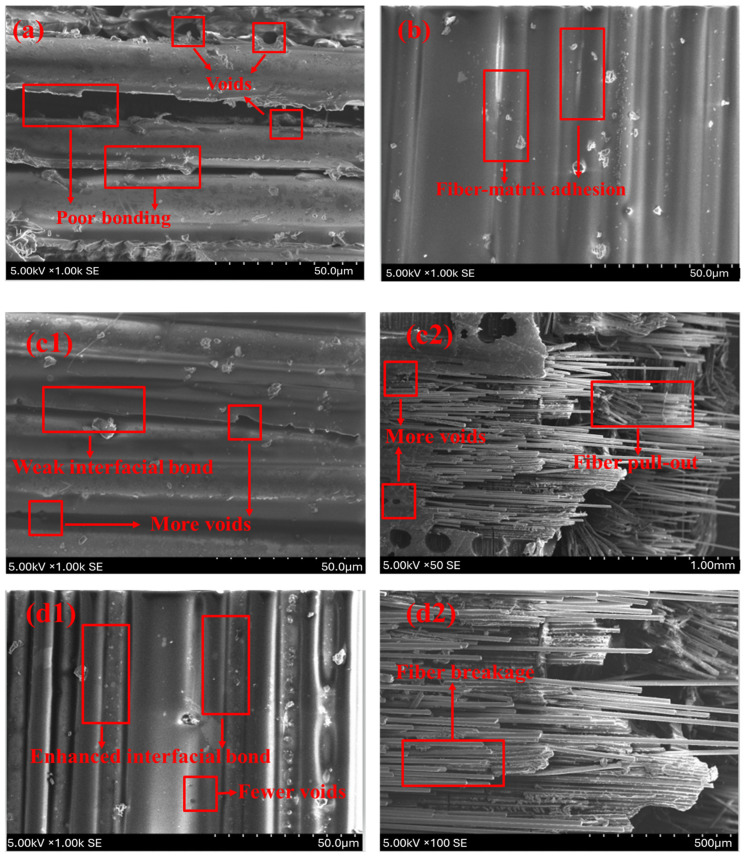
SEM of tensile-fractured specimens (**a**) J2G2; (**b**) T-J2G2; (**c1**,**c2**) F2G2; and (**d1**,**d2**) T-F2G2.

Flax/glass composites consistently outperform jute/glass composites in both tensile strength and elastic modulus. This can be attributed to the higher intrinsic mechanical properties of flax fibers compared to jute. Potassium hydroxide (KOH) treatment removes surface impurities such as lignin, hemicellulose, pectin, and waxes from natural fibers. This exposes more hydroxyl groups, which enhance interfacial bonding with the epoxy matrix [48]. Compared to NaOH, KOH is a gentler alkaline agent that causes less degradation of the cellulose backbone, preserving fiber integrity and mechanical properties. The removal of non-cellulosic components increases surface roughness and enhances mechanical interlocking with the matrix. The exposed hydroxyl (–OH) groups promote hydrogen bonding, leading to improved load transfer between fibers and the matrix. Most existing studies focus on NaOH due to its widespread availability and established effectiveness. However, recent studies highlight that KOH treatment results in comparable or even superior fiber–matrix adhesion with less structural damage. By highlighting the advantages of KOH treatment, this study contributes to the growing body of knowledge on sustainable and high-performance hybrid composites for advanced engineering applications [49]. Weak fibers with fibrillation lose their ability to effectively carry loads and transfer stress, leading to easier fiber pull-out during loading [50,51]. This behavior primarily accounts for the reduced strength and modulus observed at specific fiber loadings in composites with alkali-treated fibers. The microstructure of tensile-fractured specimens with the untreated and KOH-modified fibers obtained from the SEM is given in Figure 6.

The SEM images display the tensile-fractured surfaces of untreated and KOH-treated hybrid composites. In the untreated J2G2 sample, Figure 6a, poor fiber–matrix bonding is evident, characterised by visible voids and significant fiber pull-out. In contrast, for the treated T-J2G2 sample, Figure 6b shows enhanced fiber–matrix adhesion with fewer pull-outs, aligning with the observed increase in tensile strength and elastic modulus (58.35 MPa and 4.31 GPa, respectively). Similarly, the untreated F2G2 sample, Figure 6c1,c2 exhibits fiber pull-out and noticeable voids, indicating weak interfacial bonding. However, the treated F2G2 sample, Figure 6d1,d2 demonstrates reduced voids, improved bonding, and predominant fiber breakage instead of pull-out, supporting the substantial improvements in tensile strength (118.16 MPa) and modulus (6.5 GPa). These observations confirm that KOH treatment effectively enhances interfacial adhesion, resulting in enhanced mechanical performance, as reflected in the graph.

#### 3.2.2. Flexural Properties

**Figure 7:**
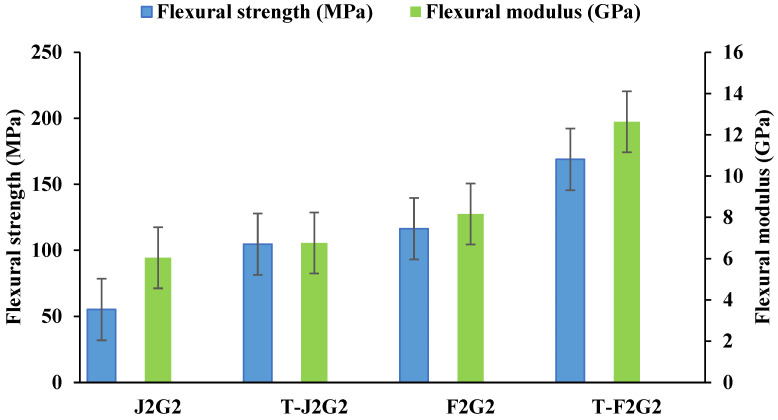
Graph showing flexural strength and flexural modulus of treated and untreated hybrid composites.

The flexural strength and modulus results shown in Figure 7 demonstrate the significant improvements achieved through KOH treatment in hybrid composites, particularly for T-J2G2 and T-F2G2, when compared to their untreated counterparts (J2G2 and F2G2). Untreated jute/glass composites (J2G2) exhibit the lowest flexural strength (55.32 MPa) and modulus (6.04 GPa), highlighting the weaker mechanical properties of jute fibers relative to flax fibers. Untreated flax/glass composites (F2G2), on the other hand, show higher flexural strength (116.43 MPa) and modulus (8.16 GPa), reflecting the intrinsic stiffness and strength of flax fibers.

KOH treatment significantly enhances the mechanical properties of the composites. Treated jute/glass composites (T-J2G2) achieve a flexural strength of 104.73 MPa, nearly double that of untreated J2G2, and a modulus of 6.76 GPa. The improvement is more pronounced in treated flax/glass composites (T-F2G2), with a flexural strength of 168.94 MPa and modulus of 12.63 GPa, indicating superior fiber–matrix bonding. The improved performance of the composites can be attributed to the KOH treatment’s ability to effectively remove non-cellulosic impurities, such as lignin, hemicellulose, and pectin, from the fiber surface. This process exposes hydroxyl (–OH) groups, enhancing hydrogen bonding and mechanical interlocking with the epoxy matrix. Furthermore, KOH treatment roughens the fiber surface, thereby improving the interfacial adhesion between the fibers and the matrix. Unlike harsher alkali treatments like NaOH, which can degrade the cellulose structure, KOH is a milder treatment that preserves the integrity of the fibers, ensuring better retention of mechanical properties. These results are also influenced by the inherent mechanical properties of flax and jute fibers, with flax being naturally stronger and stiffer than jute, leading to superior performance in flax/glass composites. The results validate the efficacy of KOH processing in improving fiber–matrix reactions, rendering it a significant method for augmenting the functionality of hybrid materials. Recent investigations [52,53] underscore the comparable advantages of the use of alkali, hence affirming its significance in enhancing the mechanical properties of natural fiber-reinforced composites.

**Figure 8:**
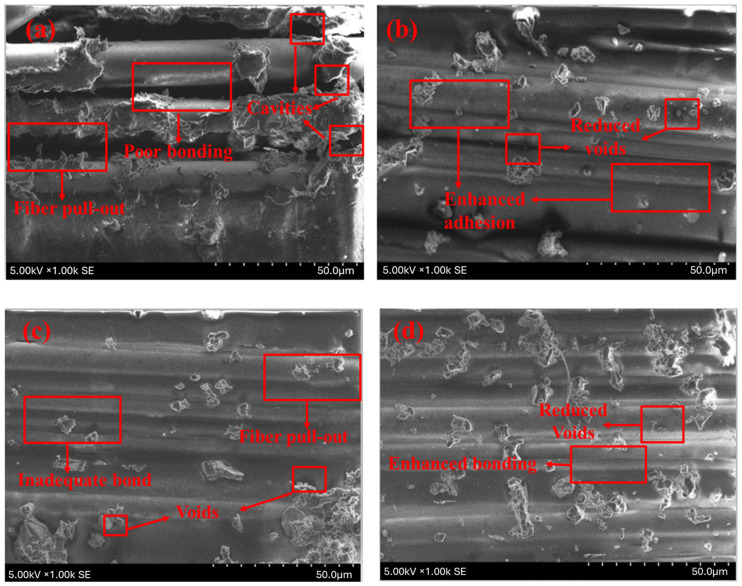
SEM of fractured flexural specimens (**a**) J2G2; (**b**) T-J2G2; (**c**) F2G2; and (**d**) T-F2G2.

The SEM micrographs depict the bending surface fractures of both unprocessed and KOH-treated hybrid composites, revealing fiber–matrix interconnections. In the unprocessed jute/glass composite materials (J2G2) seen in Figure 8a, considerable fiber pull-out and cavities are evident, signifying inadequate interfacial adhesion. In contrast, KOH-treated jute/glass composites (T-J2G2) in Figure 8b show reduced voids and enhanced fiber–matrix adhesion, correlating with the observed improvement in flexural strength (104.73 MPa). Raw flax/glass composites (F2G2) in Figure 8c demonstrate significant fiber pull-out and inadequate bonding, whereas the processed flax/glass composites (T-F2G2) in Figure 8d reveal less voids, enhanced connection, and indications of fiber breakage rather than pull-out. The morphological alterations validate the efficacy of KOH processing in eliminating contaminants and augmenting interfacial adhesion, resulting in a notable enhancement of physical properties, as evidenced by the elevated flexural strength (168.94 MPa) and modulus (12.63 GPa) for T-F2G2. These results validate the potential of KOH-treated composites for applications requiring enhanced mechanical performance.

#### 3.2.3. Impact Properties

**Figure 9:**
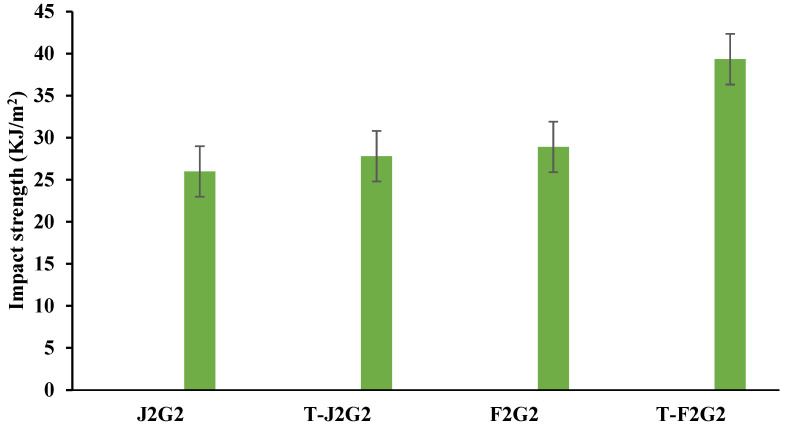
Graph shows the impact strength of treated and untreated hybrid composites.

Figure 9 with bars depicts the impact energy (KJ/m^2^) of unprocessed and KOH-treated hybrid composite materials, specifically J2G2, T-J2G2, F2G2, and T-F2G2. The unmodified jute/glass composite (J2G2) exhibits an impact capacity of 25.99 KJ/m^2^, which marginally improves to 27.8 KJ/m^2^ for the KOH-treated jute/glass composite (T-J2G2). Similarly, the untreated flax/glass composite (F2G2) exhibits a slightly higher impact strength of 28.9 KJ/m^2^ compared to J2G2. The KOH-treated flax/glass composite (T-F2G2) demonstrates a significant improvement, achieving an impact strength of 39.33 KJ/m^2^. This substantial increase highlights the effectiveness of KOH treatment in enhancing the interfacial bonding between fibers and the matrix, improving the composite’s ability to absorb and dissipate energy during impact.

The increase in impact strength for KOH-treated samples can be attributed to the removal of non-cellulosic components such as lignin, pectin, and waxes, which expose more hydroxyl (–OH) groups, leading to improved fiber–matrix adhesion. The enhanced fiber–matrix interaction minimises void formation and fiber pull-out, improving energy absorption under impact conditions.

**Figure 10:**
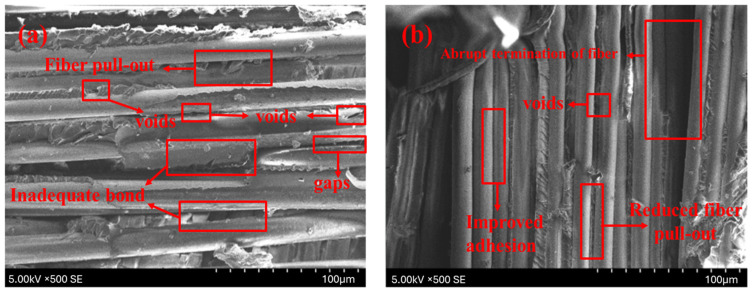

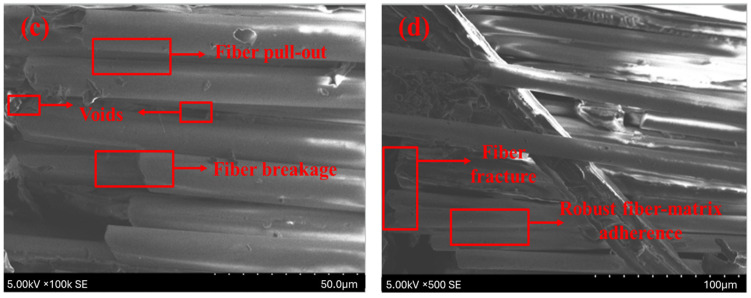
SEM of pendulum impact fractured specimens. (**a**) J2G2; (**b**) T-J2G2; (**c**) F2G2; and (**d**) T-F2G2.

The SEM images in Figure 10 illustrate the fractured surfaces of pendulum impact-tested hybrid composites, highlighting the influence of KOH treatment on fiber–matrix interaction. The untreated jute/glass composite (J2G2) in Figure 10a shows poor interfacial bonding, with noticeable fiber pull-out and gaps, indicative of weak energy absorption during impact. In contrast, the KOH-treated jute/glass composite (T-J2G2) in Figure 10b demonstrates improved adhesion, as evidenced by reduced pull-out and more fractured fibers, which correspond to the observed increase in impact strength to 27.8 KJ/m^2^. Similarly, the untreated flax/glass composite (F2G2) in Figure 10c reveals moderate fiber pull-out and voids, reflecting the relatively better performance of flax fibers due to their better mechanical properties compared to jute. The modified flax/glass composite (T-F2G2) in Figure 10d demonstrates robust fiber–matrix adherence, characterized by low fiber pull-out and primary fiber fracture, corroborating the markedly improved impact value of 39.33 KJ/m^2^. These findings validate the efficacy of KOH processing in augmenting fiber–matrix adhesion, consequently boosting the energy absorption and mechanical properties of the prepared materials.

## 4. Conclusions

This research examined the microstructural and mechanical characteristics of untreated and KOH-treated hybrid composites, which consist of jute/glass and flax/glass fibers reinforced with epoxy resin. The KOH intervention. successfully eliminated non-cellulosic contaminants, including lignin, hemicellulose, and pectin, thereby improving fiber–matrix adhesion and maintaining the dimensional stability of cellulose. The findings indicated notable enhancements in the mechanical properties of the addressed composites. KOH-treated flax/glass composites (T-F2G2) demonstrated superior mechanical properties, achieving a tensile strength of 118.16 MPa, an elastic modulus of 6.5 GPa, a bending strength of 168.94 MPa, and an impact energy of 39.33 KJ/m^2^, surpassing both addressed and non-treated jute/glass composites. The scanning electron microscope (SEM) study demonstrated enhanced interfacial connection, decreased voids, and decreased fiber pull-out in treated composite materials, thereby confirming the enhancement of mechanical strength.

The results indicate that KOH treatment is a practical and environmentally friendly approach for enhancing the functionality of organic composites with fiber reinforcement. This research presents a comparison of flax and jute as reinforcements, demonstrating that flax is a more effective fiber for uses requiring greater durability and rigidity. This study advances the fabrication of lightweight, environmentally sustainable composite materials for fundamental and industrial uses while enhancing the understanding of alkaline processes in composites made of natural fibers. The combined effect of alkaline treatment and fiber-hybridization-enhanced fiber–matrix adhesion, reduced defects, and improved stress transfer efficiency, thereby significantly contributing to the superior mechanical performance of the hybrid composites. Prospective studies may investigate the long-term durability, moisture resistance, and environmental aging of KOH-treated hybrid composites to assess their suitability for real-world applications. Additionally, this study primarily focuses on mechanical characterisation, and future studies could explore thermal, electrical, and biodegradability aspects for broader applicability in industrial sectors.

## Figures and Tables

**Table 1:** Physical and mechanical properties of flax fibers.

Properties/Features	Value
Fiber density	1.45 ± 0.02 g/cm^3^
Areal density	204 g/m^2^
Elongation at break	1.3%
Water content ^1^	5–7%
Apparent modulus	58 ± 2 GPa

^1^ Valid under the following conditions: 22 °C, 50% RH.

**Table 2:** Physical and mechanical properties of jute fibers.

Properties/Features	Value
Fiber density	1.45 ± 0.03 g/cm^3^
Areal density	233 g/m^2^
Elongation at break	1.5–2.0%
Water content ^1^	8–10%
Apparent modulus	30 ± 2 GPa

^1^ Valid under the following conditions: 22 °C, 50% RH.

**Table 3:** Physical and mechanical properties of glass fibers.

Properties/Features	Value
Fiber density	2.54 ± 0.02 g/cm^3^
Areal density	200 g/m^2^
Elongation at break	2.5%
Water content	0%
Apparent modulus	70 ± 2 GPa

**Table 4:** Araldite epoxy properties.

Properties/Features	Value
Density	1.2 g/cm^3^
Tensile strength	85–90 MPa
Tensile modulus	2.8–3.2 GPa
Tensile elongation at break	4.5–5.5
Flexural strength	120–130 MPa
Flexural modulus	2.9–3.5
Glass transition temperature	120–130 °C

**Table 5:** Details of fabricated hybrid composites.

Sample Code	Reinforcement	Reinforcement Treatment
J2G2	2 layers of jute, 2 layers of glass (J/G/J/G [0/90/90/0])	No treatment
F2G2	2 layers of flax, 2 layers of glass (F/G/F/G [0/90/90/0])	No treatment
T-J2G2	2 layers of jute, 2 layers of glass (J/G/J/G [0/90/90/0])	Alkaline with KOH
T-F2G2	2 layers of flax, 2 layers of glass (F/G/F/G [0/90/90/0])	Alkaline with KOH

J2G2, F2G2 (Untreated samples), and T-J2G2, T-F2G2 (Treated samples).

**Table 6:** Physical properties of composite preform/fibers.

Sample Code	Yarn Count (Warp)	Yarn Count (Weft)	Ends/Inch	Picks/Inch	Areal Density (g/m^2^)	Tensile Strength (Warp) (N)	Tensile Strength (Weft) (N)	Shrinkage (Warp) (%)	Shrinkage (Weft) (%)
F-BT	12.15	18.41	52.5	51.5	204	588.6	490.5	4.43	3.04
F-AT	___	___	62	58	210	294.3	259.97	0.55	0.45
J-BT	1.91	1.81	9	9	233	313.92	274.68	5.00	2.14
J-AT	___	___	12	10	315	147.15	132.44	0.35	0.27

## Data Availability

All data are contained within this article.
